# Heterogeneity of patient-reported outcome measures in clinical research

**DOI:** 10.1186/s12955-024-02282-7

**Published:** 2024-08-17

**Authors:** Jan Henrik Terheyden, Lisa Gittel, Julie Jungblut, Deanna J. Taylor, Frank G. Holz, David P. Crabb, Robert P. Finger

**Affiliations:** 1 Department of Ophthalmology, University Hospital Bonn, Ernst-Abbe-Str. 2, Bonn, 53127 Germany; 2 Department of Optometry and Visual Sciences, City, University of London, School of Health and Psychological Sciences, London, UK; 3 Department of Ophthalmology, University Hospital Mannheim and Medical Faculty Mannheim, University of Heidelberg, Mannheim, Germany

**Keywords:** Patient-reported outcome measures, Ophthalmology, Clinical trials

## Abstract

**Background:**

The use of patient-reported outcome measures (PROMs) in clinical research increases and use of heterogeneous instruments reflects how well diverse traits are captured by a medical specialty. In order to reflect the heterogeneity of current PROM use in ophthalmology, we reviewed the available literature.

**Methods:**

The medical literature database Web of Science was searched for the most cited articles in clinical ophthalmology. Titles, abstracts and full text articles were reviewed for the use of PROMs and a list of the 100 most cited articles using PROMs was obtained and stratified by year of publication.

**Results:**

A total of 1,996 articles were screened. Seventy-seven out of the 100 articles identified included one PROM, and the average number of instruments was 1.5 ± 1.1. The most widely used PROMs were the National Eye Institute Visual Function Questionnaire (33%), the Ocular Surface Disease Index (14%) and the Medical Outcomes Study Short Form (13%). A simulation analysis suggested that the distribution of PROM use in ophthalmology study did not significantly differ from a power law distribution. Twenty-two percent and fifteen percent of articles did not reference and did not specify the PROM used, respectively. This rate decreased in the more recently published articles (*p* = 0.041).

**Conclusions:**

Our data suggest that the heterogeneity of PROMs applied in ophthalmology studies is low. The selection of PROMs for clinical studies should be done carefully, depending on the research goal.

**Supplementary Information:**

The online version contains supplementary material available at 10.1186/s12955-024-02282-7.

## Background

Patient-reported outcome measures (PROMs) are increasingly used across medical disciplines [1]. Even though vision is often valued as the most important sense and visual impairment has detrimental effects on quality of life [2, 3], many generic PROMs do not include vision-related domains. For this reason, a variety of ophthalmic PROMs has been developed, measuring, for example, aspects relevant to quality of life that relate to the various domains of visual function (vision-related quality of life, VRQoL) [4].

When choosing appropriate PROMs in the context of clinical studies, face validity, i.e. overlap between the measured and the intended construct, is a key criterion and besides including generic PROMs, use of specific instruments has been recommended to detect patient-relevant changes [5, 6]. This suggests that the heterogeneity in the selection of PROMs in a medical speciality is an indicator for the level at which the selection of PROMs in a research context follows scientific recommendations. The National Eye Institute Visual Function Questionnaire (NEI-VFQ) is often cited as the most commonly used generic PROM in ophthalmology [4, 7] but the heterogeneity of PROMs used has never been systematically assessed. We have therefore investigated the level of heterogeneity of PROMs in ophthalmology in a sample of the most cited original articles.

## Methods

We performed a medical literature search for the most cited articles in the ophthalmology category of the database Web of Science™ (Clarivate, Philadelphia, Pennsylvania) on 10/01/2023, without time restrictions. A database extract of the most cited articles was downloaded and article titles, abstracts and full texts were reviewed for the use of PROMs, based on a search string recommended by the consensus-based standards for the selection of health measurement instruments (COSMIN) initiative as a guidance (Supplementary file). The measurement instruments used in the final list of articles were then identified. Inclusion criteria were original articles listed in the category ophthalmology and including structured patient-reports as one of the study outcomes. Reviews, commentaries and editorials and articles including performance-based tests, experience measures and reports by a proxy were excluded. A list of 100 most cited, eligible articles which included PROM data was compiled, as done in previous bibliometric analyses [8, 9]. This sample size is justified by the consideration of 64% of trials in the field of age-related macular degeneration using the NEI-VFQ [7], as well as a confidence level of 95% and a 10% margin of error, which suggests a sample of ≥ 89 studies.

The number of PROMs per article was summarized by mean and standard deviation and the distribution of all identified PROMs in the study cohort was analysed for heterogeneity, under the hypothesis that the use of PROMs in the identified studies followed a power law distribution, i.e. described by a mathematical function similar to *f(x)* = *ax*^*n*^, which is a common distribution for a variety of phenomena [10, 11]. Power law distributions are characterized by a small minority of instances making up the vast majority of the distribution. A power equation for the PROM dataset was estimated, including all PROMs that could be categorized, and the distribution of the PROM data was compared to iteratively simulated power law distributions, using the Kolmogorov Smirnoff test [12]. A total of 2,500 iterations was performed and the level of significance for this analysis was chosen as 0.1, which is in accordance with the recommendations for this methodology [12]. It is noted that *p*-values above 0.1 indicate the distribution of our dataset being not significantly different from the simulated power law distribution. Therefore, we retained our null hypothesis (i.e., our data follow a power law distribution) if the *p*-value was ≥ 0.1 in more than 250 iterations. Besides assessment of heterogeneity, time trends compared to the median publication year were analysed, using Fisher’s exact test and the Wilcoxon test. Here, *p*-values < 0.05 were considered statistically significant. All analyses were performed using SPSS (version 26, IBM, Armonk, NY) or R (version 4.2.2, R project, Vienna Austria).

## Results

A total number of 1,996 most cited ophthalmology publications were reviewed to identify 100 eligible articles, of which 1,206 were excluded after initial review based on the COSMIN search string and an additional 690 were excluded after applying the remaining exclusion criteria, leaving 100 articles for analysis. All excluded articles did not report any use of PROMs or were no original articles. The identified, most cited papers were published between 1991 and 2017 (median year: 2004), and were cited between 242 and 2,723 times as per January 2023. Twenty-six PROM instruments were used and the mean number of PROMs per article was 1.5 ± 1.1, which did not significantly change over time (*p* = 0.403). The majority of articles (77.0%) reported use of one single PROM. Seventy-eight (91.8%) articles included ophthalmic PROMs while 26 (30.6%) articles included generic PROMs and 19 articles (22.4%) included both, with no significant trends over time (*p* ≥ 0.250).

**Fig. 1:**
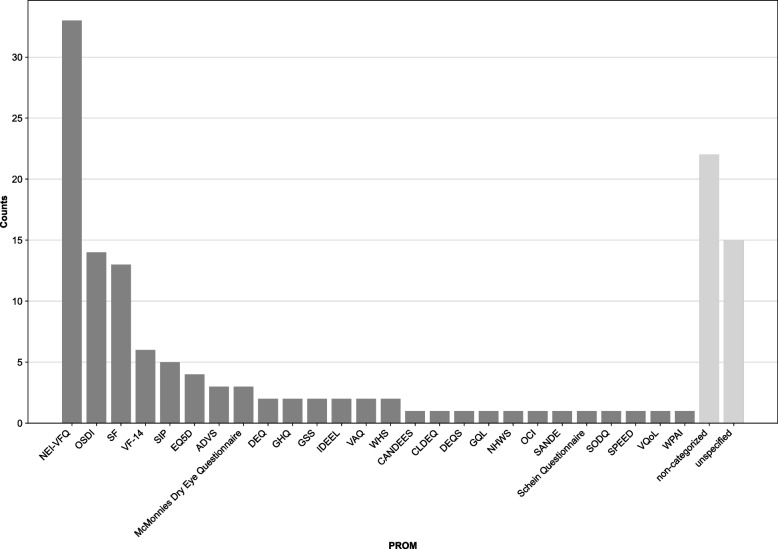
Patient-reported outcomes in the most cited ophthalmology studies. The frequency distribution of instruments follows a power law distribution. ADVS = Activities of Daily Vision Scale; CANDEES = Canada Dry Eye Epidemiology Study questionnaire; CLDEQ = Contact Lens Dry Eye Questionnaire; DEQ = Dry Eye Questionnaire; DEQS = Dry Eye-related Quality of Life scores; EQ5D = European Quality of Life 5 Dimensions version; GHQ = General Health Questionnaire; GQL = Glaucoma Quality of Life Questionnaire; GSS = Glaucoma Symptoms Scale; IDEEL = Impact of Dry Eye on Everyday Life Questionnaire; NEI-VFQ = National Eye Institute Visual Function Questionnaire; NHWS = National Health and Wellness Survey; OCI = Ocular Comfort Index; OSDI = Ocular Surface Disease Index; SANDE = Symptom Assessment in Dry Eye Questionnaire; SF = Medical Outcomes Study Short Form; SIP = Sickness Impact Profile; SODQ = Symptoms of discomfort questionnaire; SPEED = Standard Patient Evaluation of Eye Dryness; VAQ = Visual Activities Questionnaire; VF-14 = Visual Function Index 14; VQoL = Vision Quality of Life Questionnaire; WHS = Womens ‘ Health Study Questionnaire; WPAI = Work Productivity and Activity Impairment Questionnaire; unspecified = use of patient-reports stated with no further specification; non-categorized = use of patient-report described but not referenced

The NEI-VFQ was the most commonly used instrument, reported in one third of studies and more than twice as often as any other PROM. It was implemented as the only PROM in 23 studies (69.7%). Use of the NEI-VFQ was followed by the Ocular Surface Disease Index (OSDI) and the Medical Outcomes Study Short Form (SF-36 or SF-12), used in 14% and 13% of studies, respectively. Visual inspection of the histogram (Fig. 1) indicated a power law distribution and 2,484 out of 2,500 test iterations (99.4%) suggested no significant difference of the frequency distribution of the PROM dataset from a power distribution, which exceeds the threshold expected by chance by factor > 9.

A noticeable proportion of studaies used an instrument only described in the paper but not further referenced (22.0%) or not specified at all (15.0%). This trend decreased significantly in the more recent publications [since year 2004] (*p* = 0.041).

## Discussion

Our study demonstrates the use of PROMs in clinical studies follows a power law distribution, with particularly one generic vision-related quality of life instrument (NEI-VFQ) being used widely in ophthalmology studies. This finding contrasts the heterogeneity of available PROM instruments that are targeted at specific deficits and have been validated for common conditions [4]. A noticeable proportion of studies implemented PROMs not otherwise validated or used in the field. However, this trend significantly decreased over time, which implies a rise in quality standards.

The use of generic and condition-specific PROMs is widely recommended in the literature [5, 13] to ensure comparability across conditions and track changes in specific aspects. However, the majority of studies (70%) using the most common PROM in ophthalmology included only this very instrument, which contradicts the above recommendation. In contrast to this, the majority of PROMs newly developed are condition-specific or function-specific instruments [14]. The use of only a generic instrument in many of the identified studies comes with the downside of losing specificity and a risk of non-detection of patient-relevant changes [5]. Our results suggest that more efforts are needed to popularize condition-specific or function-specific PROMs and promote rigorous assessment and selection of available PROMs targeted at the specific research question, which is also supported by an evaluation of PROMs used in the context of labelling claims [15].

With a higher proportion of articles describing the use of systematically developed PROMs in more recent studies, our results suggest an improvement in the scientific process during the selection of instruments for clinical research. This aligns with initiatives promoting the correct use of PROMs, such as COSMIN [6].

Strengths of our work include its thorough methodology assessing the use of PROMs in a large set of articles and its analysis strategy. Its limitations include a possible sampling bias by only including the most cited articles in the analysis, the comparability to other medical disciplines and the use of historical data.

## Conclusions

Our study suggests that there is little heterogeneity in the use of PROMs in high-impact ophthalmology studies. Researchers are encouraged to include patient-relevant endpoints into studies but the choice of instruments should be made carefully and based on scientific rationale.

### Supplementary Information

Supplementary Material 1

## Data Availability

The datasets used and analysed during the current study are available from the corresponding author upon reasonable request.

## Abbreviations

COSMIN: COnsensus-based standards for the Selection of health Measurement INstruments
NEI-VFQ: National Eye Institute Visual Function Questionnaire (NEI-VFQ)
PROM: Patient-reported outcome measure
