# Supplementary material for: Heterogeneity of patient-reported outcome measures in clinical research
Source: Health Qual Life Outcomes. 2024 Aug 17;22:65. doi: 10.1186/s12955-024-02282-7 (PMC11330056; doi:10.1186/s12955-024-02282-7)
Supplement: Supplementary file 1 — Supplementary Material 1 [file 12955_2024_2282_MOESM1_ESM.docx]

**Heterogeneity of Patient-Reported Outcome Measures in Clinical Research**

**– SUPPLEMENT –**

**Supplementary file: Search string**

TS=("Patient Outcome Assessment") OR TS=(patient outcome assessment*) OR TS=(patient centered outcome*) OR TS=(patient centred outcome*) OR TS=(patient reported outcome*) OR TS=(prom) OR TS=(proms) OR TS=(hr pro) OR TS=(hrpro) OR TS=(health index*) OR TS=(index) OR TS=(health indices) OR TS=(health profile*) OR TS=(health status) OR ((TS=(patient) OR TS=(self) OR TS=(child) OR TS=(parent) OR TS=(carer) OR TS=(proxy)) AND ((TS=(report) OR TS=(reported) OR TS=(reporting)) OR (TS=(rated) OR TS=(rating) OR TS=(ratings)) OR TS=(based) OR (TS=(assessed) OR TS=(assessment) OR TS=(assessments)))) OR ((TS=(disability) OR TS=(function) OR TS=(functional) OR TS=(functions) OR TS=(subjective) OR TS=(utility) OR TS=(utilities) OR TS=(wellbeing) OR TS=(well being)) AND (TS=(outcome) OR TS=(outcomes) OR TS=(index) OR TS=(indices) OR TS=(instrument) OR TS=(instruments) OR TS=(measure) OR TS=(measures) OR TS=(questionnaire) OR TS=(questionnaires) OR TS=(profile) OR TS=(profiles) OR TS=(scale) OR TS=(scales) OR TS=(score) OR TS=(scores) OR TS=(status) OR TS=(survey) OR TS=(surveys)))

Source: https://www.cosmin.nl/tools/pubmed-search-filters/ (last assessed on 01/11/2023)

**Supplementary file 2: Most cited articles implementing patient-reported outcome measures in ophthalmology**

| **Authors** | **Year** | **Journal Title** | **Volume** | **Issue** | **Article Title** | **Times Cited** |
| --- | --- | --- | --- | --- | --- | --- |
|  |  |  |  |  |  |  |
|  |  |  |  |  |  |  |
|  |  |  |  |  |  |  |
|  |  |  |  |  |  |  |
|  |  |  |  |  |  |  |
|  |  |  |  |  |  |  |
|  |  |  |  |  |  |  |
|  |  |  |  |  |  |  |
|  |  |  |  |  |  |  |
|  |  |  |  |  |  |  |
|  |  |  |  |  |  |  |
|  |  |  |  |  |  |  |
|  |  |  |  |  |  |  |
|  |  |  |  |  |  |  |
|  |  |  |  |  |  |  |
|  |  |  |  |  |  |  |
|  |  |  |  |  |  |  |
|  |  |  |  |  |  |  |
|  |  |  |  |  |  |  |
|  |  |  |  |  |  |  |
|  |  |  |  |  |  |  |
|  |  |  |  |  |  |  |
|  |  |  |  |  |  |  |
|  |  |  |  |  |  |  |
|  |  |  |  |  |  |  |
|  |  |  |  |  |  |  |
|  |  |  |  |  |  |  |
|  |  |  |  |  |  |  |
|  |  |  |  |  |  |  |
|  |  |  |  |  |  |  |
|  |  |  |  |  |  |  |
|  |  |  |  |  |  |  |
|  |  |  |  |  |  |  |
|  |  |  |  |  |  |  |
|  |  |  |  |  |  |  |
|  |  |  |  |  |  |  |
|  |  |  |  |  |  |  |
|  |  |  |  |  |  |  |
|  |  |  |  |  |  |  |
|  |  |  |  |  |  |  |
|  |  |  |  |  |  |  |
|  |  |  |  |  |  |  |
|  |  |  |  |  |  |  |
|  |  |  |  |  |  |  |
|  |  |  |  |  |  |  |
|  |  |  |  |  |  |  |
|  |  |  |  |  |  |  |
|  |  |  |  |  |  |  |
|  |  |  |  |  |  |  |
|  |  |  |  |  |  |  |
|  |  |  |  |  |  |  |
|  |  |  |  |  |  |  |
|  |  |  |  |  |  |  |
|  |  |  |  |  |  |  |
|  |  |  |  |  |  |  |
|  |  |  |  |  |  |  |
|  |  |  |  |  |  |  |
|  |  |  |  |  |  |  |
|  |  |  |  |  |  |  |
|  |  |  |  |  |  |  |
|  |  |  |  |  |  |  |
|  |  |  |  |  |  |  |
|  |  |  |  |  |  |  |
|  |  |  |  |  |  |  |
|  |  |  |  |  |  |  |
|  |  |  |  |  |  |  |
